# Covalent Reversible Inhibitors of Cysteine Proteases Containing the Nitrile Warhead: Recent Advancement in the Field of Viral and Parasitic Diseases

**DOI:** 10.3390/molecules27082561

**Published:** 2022-04-15

**Authors:** Simone Brogi, Roberta Ibba, Sara Rossi, Stefania Butini, Vincenzo Calderone, Sandra Gemma, Giuseppe Campiani

**Affiliations:** 1 Department of Pharmacy, University of Pisa, Via Bonanno 6, 56126 Pisa, Italy; simone.brogi@unipi.it (S.B.); vincenzo.calderone@unipi.it (V.C.); 2 Department of Biotechnology, Chemistry and Pharmacy, DoE Department of Excellence 2018-2022, University of Siena, Via Aldo Moro 2, 53100 Siena, Italy; roberta.ibba@unisi.it (R.I.); rossi115@student.unisi.it (S.R.); butini3@unisi.it (S.B.); campiani@unisi.it (G.C.)

**Keywords:** nitrile, warhead, cysteine/serine/threonine protease, virus, protozoan parasite, covalent inhibition

## Abstract

In the field of drug discovery, the nitrile group is well represented among drugs and biologically active compounds. It can form both non-covalent and covalent interactions with diverse biological targets, and it is amenable as an electrophilic warhead for covalent inhibition. The main advantage of the nitrile group as a warhead is mainly due to its milder electrophilic character relative to other more reactive groups (e.g., -CHO), reducing the possibility of unwanted reactions that would hinder the development of safe drugs, coupled to the ease of installation through different synthetic approaches. The covalent inhibition is a well-assessed design approach for serine, threonine, and cysteine protease inhibitors. The mechanism of hydrolysis of these enzymes involves the formation of a covalent acyl intermediate, and this mechanism can be exploited by introducing electrophilic warheads in order to mimic this covalent intermediate. Due to the relevant role played by the cysteine protease in the survival and replication of infective agents, spanning from viruses to protozoan parasites, we will review the most relevant and recent examples of protease inhibitors presenting a nitrile group that have been introduced to form or to facilitate the formation of a covalent bond with the catalytic cysteine active site residue.

## 1. Introduction

Around 2.3% of currently approved drugs contain a nitrile group [1]. This percentage increases to 4.3–4.5% when considering biologically active compounds or drug-like small molecules, such as those reported in the ChEMBL or Zinc databases [1,2]. The nitrile functional group is formed by an sp-hybridized C atom linked to a N atom, with the latter presenting a lone pair. Relevant features of the nitrile group are its linear shape, its electron-withdrawing properties, and the electrophilic character of the sp C atom. The nitrile group can contribute to improving drug–target affinity through the formation of covalent or non-covalent interactions [3]. Among the non-covalent interactions, the possibility of forming H bonds or polar interactions inside specific pockets are the most relevant [2]. Regarding the covalent interactions, thanks to its electrophilicity, it can reversibly react with active-site Ser or Cys residues to form (thio)imidate adducts through the Pinner reaction [4,5]. Moreover, its electron-withdrawing character can activate neighboring double bonds toward Michael additions by cysteine residues [5].

Proteases are an important class of drug targets that are heavily exploited in drug discovery and design for the identification of several drugs useful in different pathologies. In the frame of infectious diseases, proteases play crucial roles in the life cycle stages and have been investigated as drug targets for a number of infective agents, especially protozoan parasites, such as *Plasmodium falciparum*, *Toxoplasma gondii*, *Trypanosoma cruzi*, *Trypanosoma brucei*, *Leishmania* spp., and others [6,7,8,9,10,11]. The proteolytic activity of host and virally encoded proteases is also important in the replicative cycles of pathogenic viruses [6,12]. Aspartic protease inhibitors are the mainstays of the therapeutic arsenal against the human immunodeficiency virus (HIV) infection, while, in recent years, a tremendous effort aimed at the identification of inhibitors of the serine protease of the hepatitis C virus (HCV) virus culminated in the discovery of boceprevir and telaprevir [13]. Proteases can be distinguished in two main groups based on their catalytic mechanism. The first mechanism involves the activation of a water molecule that is then able to perform a nucleophilic attack at the carbonyl carbon of the peptide bond that becomes hydrolyzed. The resulting tetrahedral intermediate then collapses, releasing the reaction products, namely, the amine and the carboxylic acid. Examples of proteases displaying this mechanism are aspartic proteases and metalloproteases where the water molecule is activated by the aspartic acid residues or by a bound metal, respectively [13]. The second mechanism involves the use of a nucleophilic residue in a catalytic triad or dyad that performs a nucleophilic attack toward the carbonyl carbon of the peptide bond to be hydrolyzed. Upon such a reaction step, a covalent bond is formed among the catalytic residue of the protease and the substrate protein, and the first half of the product is, thus, released. The resulting covalent acyl-enzyme intermediate is then hydrolyzed by a molecule of water to complete the catalytic cycle, and the second half of the product is released. After this last reaction step, the free enzyme is regenerated and becomes ready for another catalytic cycle. Examples of proteases that adopt the above-described mechanism are serine, threonine, and cysteine proteases [13]. The approaches used for the design of protease inhibitors exploit these differences in the mechanisms of hydrolysis. Aspartic protease inhibitors are, for example, designed following the transition-state analogue approach, while the design of cysteine and serine protease inhibitors is based on substrate analogues in which the hydrolyzable peptide bond is replaced by an electrophilic warhead that covalently attacks the catalytic residue [13]. As a consequence, serine and cysteine proteases are a class of drug targets that could greatly benefit from the features of the nitrile group as a warhead for the design and discovery of innovative and effective drugs.

**Figure 1:**
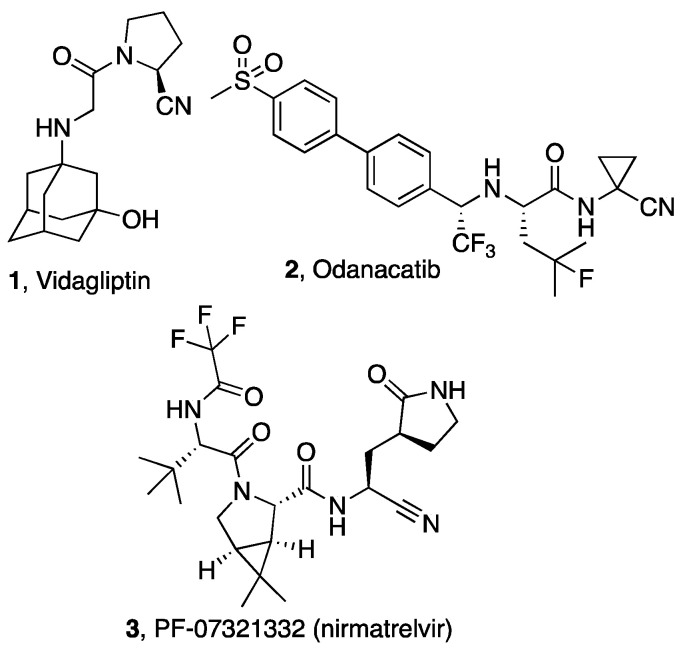
Structures of nitrile-containing drugs (**1**,**3**) and clinical candidate (**2**).

In the field of drug discovery, the increasing attention to nitriles as electrophilic warheads is mainly due to their milder electrophilic character relative to other more reactive groups (e.g., -CHO), reducing the possibility of unwanted reactions that would hinder the development of safe drugs. The structures of selected drugs (or experimental drugs) in which the nitrile group forms covalent bonds are reported in Figure 1. The nitrile group of the antidiabetic drug vidagliptin (**1**) engages the catalytic serine of dipeptidyl peptidase-4 (DPP-4), a serine protease involved in the control of incretine levels [14]. In the field of cysteine protease inhibitors, odanacatib (**2**) is an experimental drug bearing a nitrile group whose structural features and design strategy have also been taken into consideration for the discovery and optimization of small-molecule nitrile-based inhibitors of cysteine proteases of various infective pathogens. Just a few months ago, nirmatrelvir (PF-07321332) (**3**) was added to the set of nitrile-containing drugs as an inhibitor of the Mpro cysteine protease of SARS-CoV-2, the causative agent of COVID-19 [15,16].

The role of the nitrile group in medicinal chemistry has been highlighted in some reviews focused on nitrile-containing drugs [1,2,3], while several reviews cover different aspects of covalent inhibition of protein targets in the field of drug discovery [4,5,17,18]. Here, we will provide an overview of the most recent studies focusing on the nitrile group that have been published in the last years. Due to the importance of the nitrile group as a mild and selective warhead and its potential applications in the field of drug discovery, as highlighted above for current and emerging infectious diseases, here, we will survey protease inhibitors presenting a nitrile group that has been introduced to form or to facilitate the formation of a covalent bond with the catalytic cysteine active site residues. This overview will take into consideration inhibitors of proteases relevant for the life cycle or replicative cycle of infective pathogens, such as viruses and protozoan parasites, and will cover the literature published in the last 5 years.

## 2. Inhibitors of Viral Cysteine Proteases

Enterovirus 71 (EV71) belongs to the Picornavirus family of viruses. It causes neurological diseases in adults and the hand, foot, and mouth disease in children. Outbreaks of EV71 have emerged as a global health issue due to severe neurological complications leading to fatal encephalitis in a relevant number of cases. The viral genome, constituted by a single-stranded RNA molecule, is translated into a large polyprotein inside the host cell. The virally encoded protease 3Cpro plays a crucial role in the proliferation of this virus and represents a promising drug target for the design of antiviral drugs [19,20].

**Figure 2:**
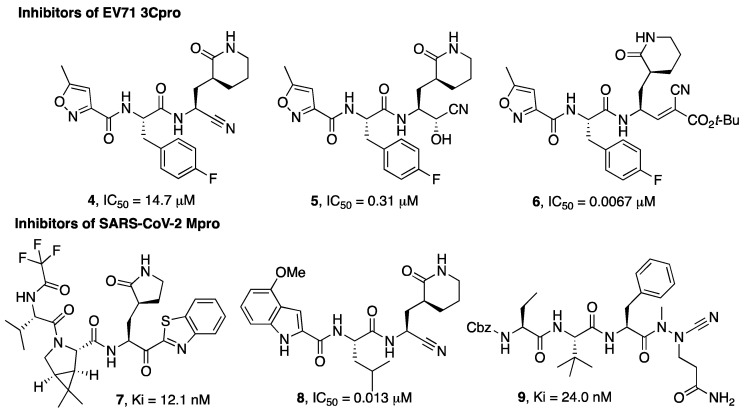
Chemical structures of viral cysteine proteases inhibitors **4**–**6** (EV71 3Cpro) and **7**–**9** (SARS-CoV-2 Mpro).

Wang et al. investigated different warheads in order to improve the stability and selectivity of a previously developed set of inhibitors of the 3Cpro enzyme of EV71 that were originally designed with an aldehyde warhead [21,22]. They introduced a panel of warheads, such as nitrile, ketoamide, alcohol, hydroxyl amide, doubly activated olefins, and hydroxy-nitrile groups, as an alternative to the aldehyde group. In this case, the nitrile warhead led to a moderately active inhibitor (**4**), while an α-hydroxynitrile gave better selectivity toward cysteine vs. serine protease and cell-based activity (inhibitor **5**, Figure 2). An X-ray crystal structure of the enzyme-bound inhibitor revealed a non-covalent binding mode [22].

A different example of a warhead was introduced in compound **6**. The electron-withdrawing properties of the cyano group were exploited for the preparation of double-activated olefins that are able to function as Michael acceptors upon reaction with the cysteine active site residue of EV71 3Cpro. The cyano group, which, in this case, does not form a covalent bond with the active site cysteine but contributes to increasing the reactivity of the olefin carbon, provided more selective enzyme inhibitory activity coupled to a better and broad-spectrum activity on cell-based assays (**6**) [23,24].

MPro is a cysteine protease with an important role in the SARS-CoV-2 replicative cycle, and since the SARS-CoV-2 epidemic outbreak in 2019, intense research efforts have been devoted by scientists in the search for MPro inhibitors [25,26,27]. These efforts culminated with the discovery of the drug Paxlovid, which is a combination of the Mpro inhibitor nirmatrelvir (**3**, Figure 1) with the metabolic booster ritonavir. Nirmatrelvir (**3**) was developed based on a structure-based optimization approach aimed at optimizing the interaction with the S1 and S2 pockets and, at the same time, monitoring the solubility and selected pharmacokinetic properties of the designated hits. The selection of **3** to be advanced in preclinical optimization over the benzothiazole analogue **7** was mainly supported by improved water solubility and a lower propensity to epimerization at the α-carbon, attributable to the presence of the cyano group instead of the benzothiazole moiety [15].

The X-ray co-crystal structures of SARS-CoV-2 Mpro in complex with nirmatrelvir confirmed the formation of a reversible covalent Cys145 adduct with the nitrile substituent [15,16]. It is worth mentioning that this inhibitor has a similar efficacy toward the recently emerged SARS-CoV-2 variants [28].

**Figure 3:**
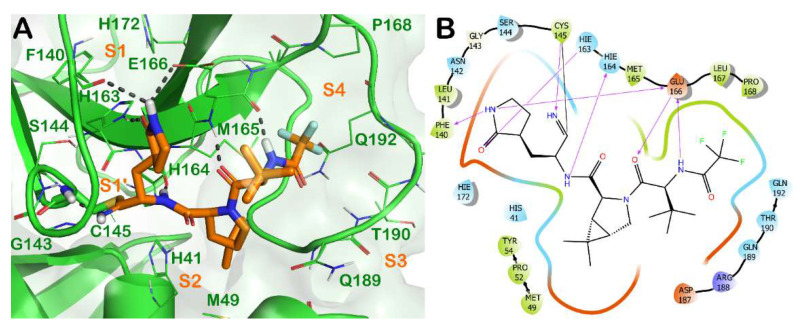
(**A**) Crystal structure of SARS-CoV-2 Mpro (green cartoon) in complex with the approved drug nirmatrelvir (**3**) (orange sticks) (PDB ID 7VH8). Interacting residues within the binding site are represented by lines, and the covalent bond established by Cys145 is represented by sticks. H bonds are illustrated by grey dotted lines. For the sake of clarity, water molecules are removed, and the complex was treated by the protein preparation wizard module available in Maestro (Maestro, Schrödinger LLC, release 2020-3). The picture was generated by PyMOL (The PyMOL Molecular Graphics System, v1.8; Schrödinger, LLC, New York, NY, USA, 2015). (**B**) Detailed interaction diagram of nirmatrelvir within the Mpro binding site, as provided by the ligand interaction diagram available in Maestro (Maestro, Schrödinger LLC, release 2020-3).

Briefly, the crystal structure of Mpro in complex with nirmatrelvir (**3**) showed a full occupancy of the ligand within the active site of the enzyme, targeting the S1-S4 subsites, as illustrated in Figure 3. The nitrile functional group interacts at the S1′ subsite, forming a covalent adduct with the reactive Cys145 of the enzyme. In addition, H bonds with the backbone of Cys145 and His164 are evident. The pyrrolidinone moiety strongly targets the S1 subsite, establishing a series of H bonds with the backbone of Phe140 and the sidechains of Glu166 and His163. The azabicyclo moiety interacts at the S2 subsite by hydrophobic contacts, while the N-terminal moiety of the molecule can establish a series of H bonds with the backbone of Glu166. The portion bearing the trifluoro function targets residues at the S3 and S4 subsites, forming hydrophobic contacts.

In the study by Bai et al. [29] a series of inhibitors typified by **8** (Figure 2) are described and, also in this study, the nitrile warhead was pointed out as a good alternative to the aldehyde warhead to achieve activity against the Mpro enzyme. Importantly, this study reports the selectivity of the compounds bearing a nitrile warhead vs. human cathepsins B, S, and L, suggesting that the designed compounds bearing a nitrile warhead were significantly less active in inhibiting cathepsins S and L than the hydroxymethylketon warhead, even though further studies are needed to support this observation.

A different warhead has been described by Bridenbach and colleagues [30]. In their search for potent SARS-CoV-2 Mpro inhibitors, two classes of compounds were reported, one of them being based on an azanitrile warhead (**9**). The authors also confirmed that these azanitrile inhibitors were able to covalently interact with the Mpro catalytic cysteine moiety.

## 3. Inhibitors of Protozoan Parasite Proteases

### 3.1. Inhibitors of Cruzain (Cz) from Trypanosoma cruzi

Cruzain (Cz) is the major cysteine protease of *T. cruzi* [31], the causative agent of Chagas disease, whose therapy is mainly based on the use of two drugs: benznidazole and nifurtimox. Both these drugs are responsible for serious side effects and are ineffective against the chronic stage of the disease [32]. Innovative therapeutic approaches are urgently needed for the treatment of this disease that affects one million people worldwide, with major diffusion in Latin American [33]. Cz has been explored for several years as a drug target, and nitrile-based inhibitors have been deeply investigated [31]. The most recent examples are reported below.

**Figure 4:**
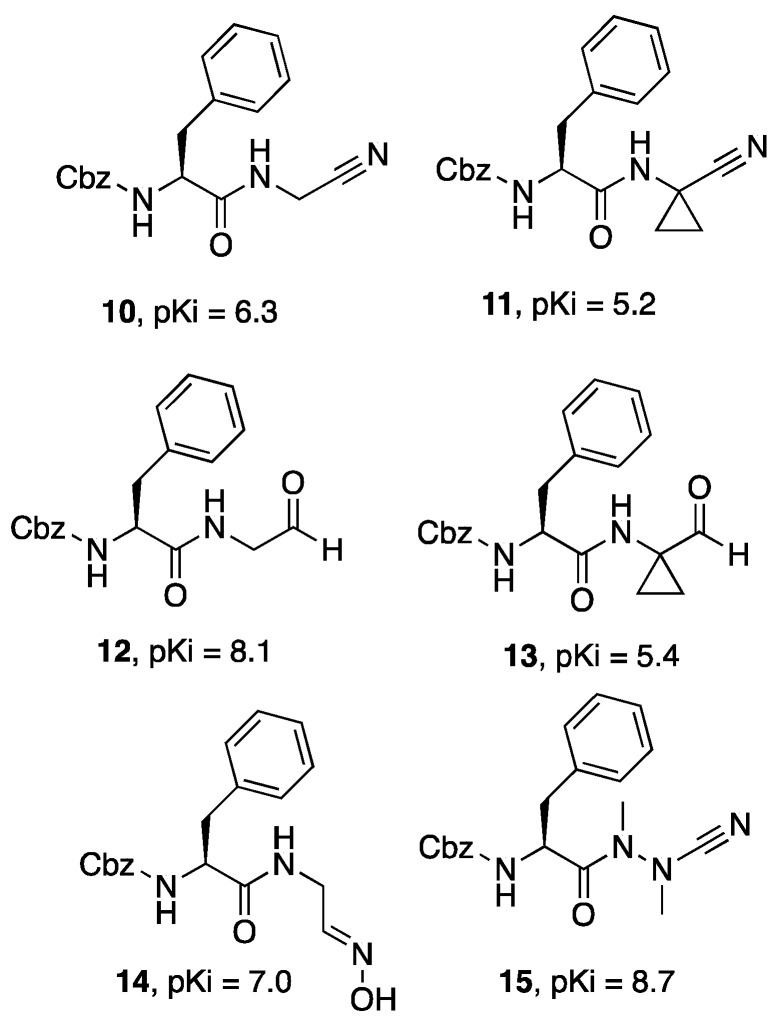
Exploration of different warheads in dipeptidic Cz inhibitors **10**–**15**.

Starting from a series of small dipeptidic derivatives (**10**, **11**, Figure 4), Silva and colleagues [34] studied the effect of replacing the nitrile warhead with alternative electrophilic moieties and evaluated their effects on Cz. Even though the original nitrile derivatives showed interesting inhibitory potencies against Cz, it was proven that the nitrile group could be productively replaced by aldehyde (**12**, **13**), oxyme (**14**), and azanitrile (**15**) functional groups. The aldehyde derivative was later demonstrated to be potently active in vitro against *T. cruzi* (Y and Tulahuen strains), showing it to be the best trypanocidal agent of the whole series [34].

**Figure 5:**
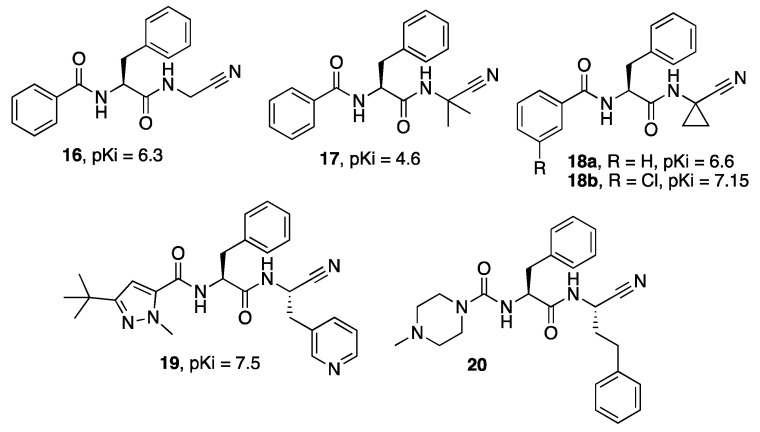
P2/P3-optimized dipeptidic inhibitors (**16**–**20**) of Cz.

Variously functionalized dipeptidyl nitriles have been reported as Cz inhibitors, and their binding mode and thermodynamics have been studied in depth, both experimentally and in silico [35,36,37,38]. For compounds **16**, **17**, and **18a,b** (Figure 5), ITC determination showed that the mechanism of binding of these inhibitors to Cz is driven by favorable enthalpy contributions coupled with the detrimental contribution of the entropy difference [36,37]. Contrastingly, for inhibitor **19,** both enthalpy and entropy are favorable to the Gibbs binding energy, thus overcoming the enthalpy–entropy compensation [38]. Moreover, a computational analysis of the reaction mechanism with the active cysteine/histidine dyad for **16**, **17** and **18a** suggested a concerted mechanism with a proton transfer from His162 to the nitrile N and the nucleophilic attack of the negatively charged Cys25 on the C atom of the nitrile moiety [37]. The same mechanism was also confirmed for the nitrile-based inhibitor **20**, which showed a different substitution pattern [39].

**Figure 6:**
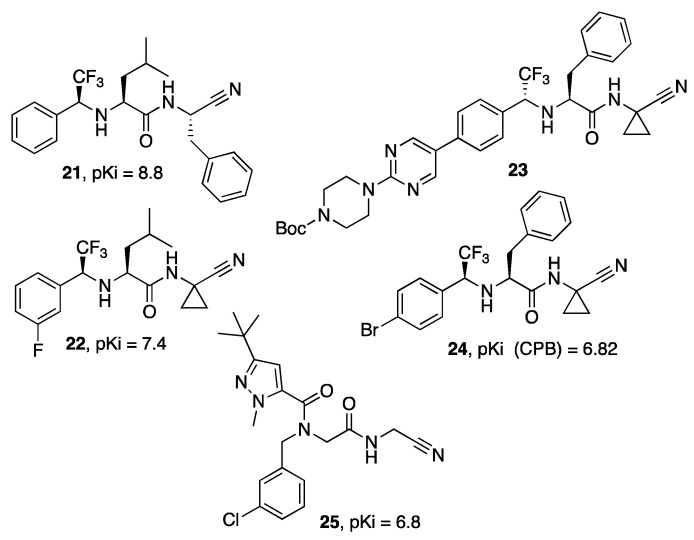
Structure of P2-trifluoroethylamine inhibitors (**21–24**) and the peptoid derivative (**25**).

Different nitrile-based Cz inhibitors in which one of the peptide bonds is replaced by a trifluoroethylamine have been reported, such as those reported in Figure 6 (**21**–**23**), and showing different degrees of selectivity toward relevant human cathepsins [40,41,42]. An investigation of the biological activity of the most promising P2-trifluorethylamine-containing inhibitors revealed a selective cytotoxic activity against the trypomastigotes and intracellular amastigotes [41]. On a related series of compounds, inhibitor **24** also showed an interesting activity against the cysteine protease B (CPB) of *Leishmania* spp. [43].

Alves et al. [44] explored the peptoid scaffold for the inhibition of cruzain. The nitrile-based peptoids typified by **25** showed a general decrease in the affinity for their target but were generally more selective vs. human cathepsins.

### 3.2. Inhibitors of Rhodesain (RD) from Trypanosoma brucei

Human African trypanosomiasis (HAT), also defined as sleeping sickness, is a parasitic disease that mainly affects sub-Saharan Africa. The parasite responsible for the disease is *Trypanosoma brucei*, for which two subspecies are known: *T. b. gambiense* and *T. b. rhodesiense*, leading to different symptomatology. The cysteine protease of *T. brucei*, namely rhodesain (RD), has been explored as a drug target through the design and synthesis of nitrile-based peptidomimetics [8,45].

**Figure 7:**
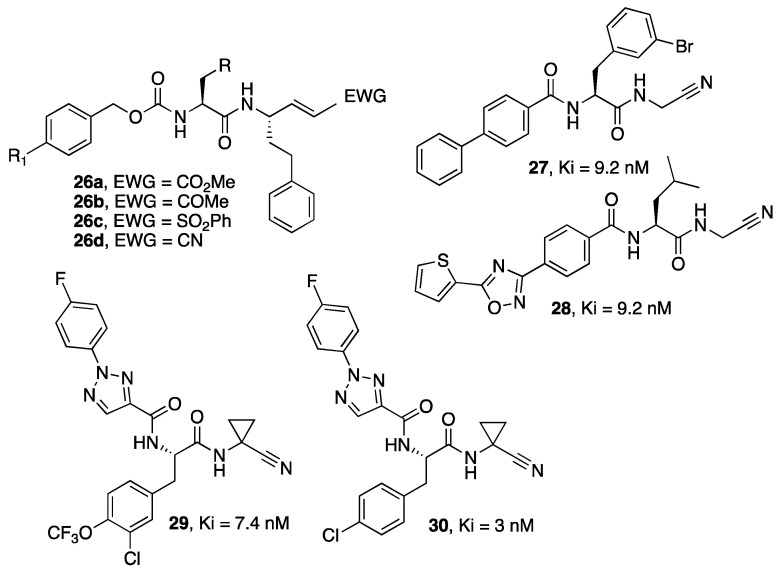
Structures of RD inhibitors (**26**–**30**).

Previti et al. [46] designed and synthesized a series of peptide-based inhibitors of RD, foreseeing the presence of Michael acceptors and exploring different activators of the olefin double bond, such as ester, nitriles, methyl ketone, and sulfone (**26a**–**d**, Figure 7). In this context, the relative inhibitory activity against RD followed the pattern ketones >> esters > sulfones > nitriles, with the nitrile-based derivatives as the least potent compounds.

The nitrile group was used as a warhead in the RD inhibitors described by Schirmeister et al. [47] (**27**,**28**), also characterized by selectivity over human cathepsins and in a series of dipeptides designed by Giroud et al. [48] (typified by **29** and **30**) that showed not only potent and selective activity against RD but also potent activity against the parasite in vitro and efficacy in vivo as well as appropriate pharmacokinetic properties.

### 3.3. Inhibitors of Cysteine Protease B from Leishmania *spp.*

Leishmaniasis is a vector-borne parasitic disease with a widespread distribution in tropical and subtropical countries, including the Mediterranean region. It has been reported that from 700,000 to 1 million new cases of leishmaniasis occur annually, while the chemotherapy for this infection is largely inadequate [49,50]. An attractive biological target for therapeutic intervention in the treatment of leishmaniasis has been identified in the cysteine protease B (CPB) expressed by this pathogenic protozoan.

**Figure 8:**
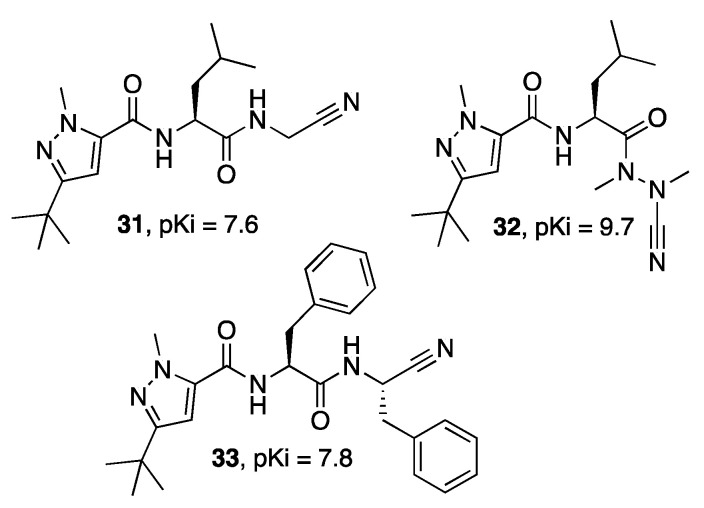
Structures of cysteine protease B inhibitors (**31**–**33**).

**Figure 9:**
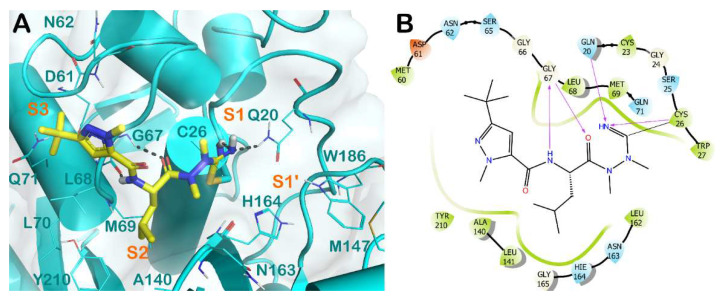
(**A**) Crystal structure of *Lm*CPB (cyan cartoon) in complex with **32** (yellow sticks) (PDB ID 6P4E). Interacting residues within the binding site are represented by lines, and the covalent bond established by Cys26 is represented by a stick. H bonds are illustrated by grey dotted lines. For the sake of clarity, water molecules are removed, and the complex was treated by the protein preparation wizard module available in Maestro (Maestro, Schrödinger LLC, release 2020-3). The picture was generated by PyMOL (The PyMOL Molecular Graphics System, v1.8; Schrödinger, LLC, New York, NY, USA, 2015). (**B**) Detailed interaction diagram of compound **32** within the *Lm*CPB binding site, as provided by the ligand interaction diagram available in Maestro (Maestro, Schrödinger LLC, release 2020-3).

Among the nitrile-containing inhibitors, compound **31** (Figure 8) also showed an interesting activity against CPB of *L. mexicana*, and optimization of the potency against this enzyme was achieved by replacing the nitrile warhead with an azanitrile group, leading to inhibitor **32,** whose crystal structure has been solved [51]. The complex of *Lm*CPB and derivative **32** is reported in Figure 9. The derivative bearing a nitrile as an electrophilic group can interact with the catalytic residue Cys26 at the S1 subsite, forming, in addition to an H bond, a covalent adduct that precludes the normal function of the enzyme. Moreover, the molecule interacts at the S1′ subsite, H binding Gln20, and at the S1 subsite, establishing a H-bond with the backbone of Gly67. The lipophilic portion of the derivative **32** forms a strong network of hydrophobic contacts with residues belonging to the S2 and S3 subsites.

**Figure 10:**
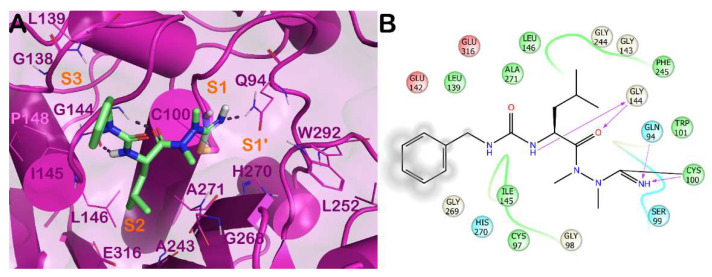
(**A**) Crystal structure of *Sm*CB1 (magenta cartoon) in complex with an azanitrile covalent inhibitor (light green sticks) (PDB ID 6YI7). Interacting residues within the binding site are represented by lines, and the covalent bond established by Cys100 is represented by a stick. H bonds are illustrated by grey dotted lines. For the sake of clarity, water molecules are removed, and the complex was treated by the protein preparation wizard module available in Maestro (Maestro, Schrödinger LLC, release 2020-3). The picture was generated by PyMOL (The PyMOL Molecular Graphics System, v1.8; Schrödinger, LLC, New York, NY, USA, 2015). (**B**) Detailed interaction diagram of a covalent inhibitor within the *Sm*CB1 binding site, as provided by the ligand interaction diagram available in Maestro (Maestro, Schrödinger LLC, release 2016).

Furthermore, azanitrile derivatives have also been demonstrated to be effective in inhibiting proteases from different parasitic agents, such as cathepsin B1 (CB1) from *Schistosoma mansoni*. The enzyme is closely related to the CPB from *L. mexicana*, sharing a similar 3D arrangement. Accordingly, the binding mode of a covalent inhibitor bearing a nitrile function within *Sm*CB1 (Figure 10) is similar to that observed for an azanitrile inhibitor within *Lm*CPB (Figure 9). The nitrile portion forms a covalent adduct with the reactive Cys100, in addition to a series of contacts with Gln94 and Cys100 at the S1′ and S1 subsites. Further H bonds with the backbone of Gly144 are detectable. The lipophilic portions of the molecules target residues at the S2 and S3 subsites by a strong network of hydrophobic contacts.

Inhibitor **33** (Figure 8) was characterized as the most potent inhibitor of LmCPB by Cianni et al. [35] in an ample study in which structure–activity relationships for the inhibition of cruzain and *Lm*CPB and selectivity vs. a panel of human cathepsins (CatB, CatK, CatL, and CatS) were investigated through the synthesis of a series of 26 new compounds.

### 3.4. Apicomplexa Parasites: Toxoplasma gondii and Plasmodium falciparum

*Toxoplasma gondii* is a neurotrophic protozoan parasite that exists in two principal forms, the tachyzoite and bradyzoite, this latter form being responsible for the chronic infection during which cysts form within the central nervous system (CNS) and muscle tissue to establish a life-long infection. *T. gondii* cathepsin protease L (TgCPL) is critical to parasite survival during the chronic phase of infection [52,53]. So, the development of effective and brain-penetrant inhibitors could lead to a great advance in the chemotherapy of this parasitic infection, which is currently ineffective against the chronic form.

**Figure 11:**
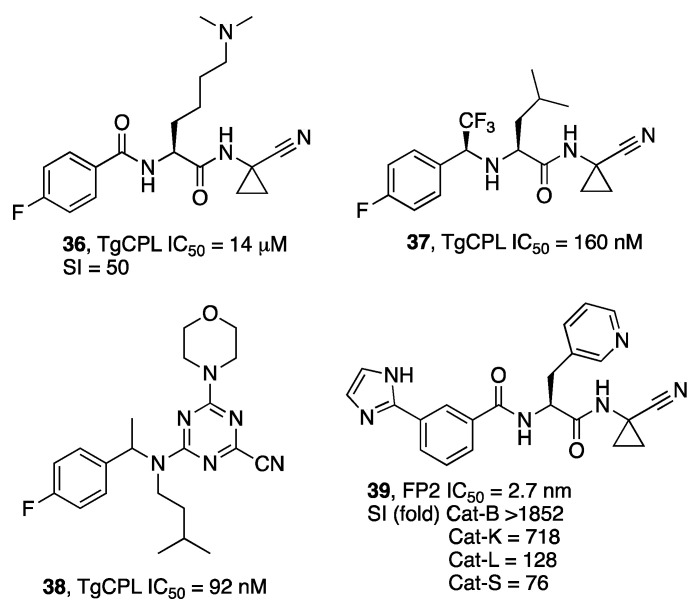
Structures of *Tg*CPL and FP2 inhibitors (**34**–**37**).

Zwicker et al. [54] discovered potent inhibitors of TgCPL. In their study, they found that selectivity over human cathepsins could be achieved by introducing basic substituents at P2, even though a decrease in potency was observed (**34**, Figure 11). Moreover, potent inhibitors were discovered through an in-depth analysis of the SARs, and some of the compounds showed the important characteristic of being brain-penetrant, even with a rapid clearance in vivo (**35**). In a further development of the work, the morphing of the peptidic scaffold with a triazine resulted in the synthesis of compounds typified by inhibitor **36** that achieved good exposure in the brains of mice after IP dosing and showed efficacy in an in vitro model of bradyzoite stage parasites [55].

Falcipain-2 (FP2) is an aspartic protease that is important in the erythrocytic life-cycle stage of *P. falciparum*, the causative agent of malaria, an infective disease with ample diffusion in tropical and sub-tropical areas of the world and responsible for a high number of deaths and new cases each year [56]. Few studies have been reported in the last years describing new inhibitors. One of these is focused on the investigation of the SAR of nitrile-based inhibitors focused on obtaining selectivity against a panel of human cathepsins [57]. Although the >100-fold selectivity that the authors set out as the main goal was not completely fulfilled, inhibitors typified by **37** showed remarkable selectivity. As observed for TgCPL, a mildly basic substituent at P2 could affect the selectivity. An in silico study suggested that the formation of a water-bridged H bond at the bottom of the S2 sub-site could be responsible for the observed selectivity [58].

## 4. Conclusions and Future Perspectives

In the last decades, especially before the 1990s, the perception of many scientists and of the pharma industry regarding drugs that are able to inhibit or bind their targets through a covalent (reversible or irreversible) mechanism was characterized by a general skepticism regarding the practical utility of such compounds. This initial skepticism seemed not to be well justified given the ample number of examples of drugs used in therapy that act through a covalent mode of action and exemplified by beta-lactam antibiotics and aspirin, to cite some the most widely used and safe drugs. A seminal article by Singh and colleagues highlighted all the pharmacological advantages of covalent vs. non-covalent drugs and many of the challenges linked to such a mechanism in the field of drug design [59]. Since then, the trend that discouraged the use of covalent inhibition in drug development has been somehow reversed, and now covalent drugs, and the approach of targeted covalent inhibition, especially for obtaining specificity and selectivity toward challenging drug targets, such as the kinases, has led to several new drugs and clinical candidates.

In the field of protease enzymes, a covalent mode of action is the most advantageous for the inhibition of the class of proteases whose mechanism of action is based on the formation of an enzyme acyl intermediate, such as those presenting activated cysteine, threonine, or serine residues in the catalytic triad (or dyad in some cases). Due to this specific mechanism of hydrolysis, the introduction of reactive electrophilic moieties is a well-established design approach for the preparation of potent inhibitors. However, the path from biologically active compounds to actual drugs needs a finely balanced reactivity in order to allow the warhead to selectively and specifically react with the active site residue of the target enzyme, avoiding non-specific reactions with free -OH and -SH groups exposed on the surfaces of proteins or other biological components. The nitrile group, with its mild electrophilic character, dimensions, and ease of chemical synthesis for its introduction in peptidic and non-peptidic scaffolds, represents a good alternative to ketoamides, aldehydes, and other reactive groups. In the specific field of proteases that are important for protozoan parasite life cycle stages, again, the stability of the nitrile group could be more advantageous for the drug design approaches toward these pathogens. The intracellular localization of most of these pathogens makes the issue of stability and selective reactivity indispensable for the design of such drug candidates. Overall, the nitrile group represents a useful warhead for the covalent reversible inhibition of cysteine and serine proteases, and it should be taken into consideration for the design of future drugs and drug candidates.

## Data Availability

Not applicable.
